# Shallow conduit dynamics fuel the unexpected paroxysms of Stromboli volcano during the summer 2019

**DOI:** 10.1038/s41598-020-79558-7

**Published:** 2021-01-11

**Authors:** Marco Viccaro, Andrea Cannata, Flavio Cannavò, Rosanna De Rosa, Marisa Giuffrida, Eugenio Nicotra, Maurizio Petrelli, Gaia Sacco

**Affiliations:** 1 Dipartimento di Scienze Biologiche, Geologiche e Ambientali, Università degli Studi di Catania, Corso Italia 57, 95129 Catania, Italy; 2 Istituto Nazionale di Geofisica e Vulcanologia – Sezione di Catania, Osservatorio Etneo, Piazza Roma 2, 95125 Catania, Italy; 3 Dipartimento di Biologia, Ecologia e Scienze della Terra, Università della Calabria, Ponte Pietro Bucci, 87036 Arcavacata di Rende, Italy; 4 Dipartimento di Fisica e Geologia, Piazza dell’Università, Università degli Studi di Perugia, 06123 Perugia, Italy

**Keywords:** Geochemistry, Geophysics, Petrology, Volcanology

## Abstract

Open conduit basaltic volcanoes can be potentially hazardous as the eruptive activity may turn suddenly from a steady state to highly explosive. Unexpected changes in explosion intensity are recurrent at Stromboli volcano, where major explosions and large-scale paroxysms sometimes break off the ordinary, Strombolian activity with little or no warning. Two powerful paroxysmal eruptions took place at Stromboli volcano during the summer 2019, causing widespread fires, consistent damages across the island, injuries and one fatality. Prediction of similar events is really challenging for the modern volcanology, though models propaedeutic to early-warning monitoring systems are not properly assessed yet in many volcanoes worldwide. Here, we present a multi-parametric study that combines petrological and geophysical data to investigate processes generating the two paroxysms. The time information derived by Li enrichments in plagioclase crystals correlates with tilt time series derived by seismometers installed on the island, highlighting the dominant role of shallow conduit processes in triggering the 2019 paroxysmal activity. Our dataset conceives a mechanism of gas slug formation and fast upward migration that finally triggered the eruptions in very limited times. The proposed model questions our capability to forecast such kind of paroxysms in times that are rapid enough to allow mitigation of the associated risk.

## Introduction

Mitigation of the risk associated to potentially dangerous eruptions worldwide is chiefly based on the recognition of eruption precursors. Most explosive, silicic stratovolcanoes may rest for thousands of years before suddenly awake with catastrophic eruptions (e.g., Mt. St. Helens^1^, Soufrière Hills^2^, Vesuvius^3^). Open-conduit basaltic volcanoes, while acknowledged for a dominant effusive activity, may disclose a highly explosive behavior likewise, generating paroxysmal eruptions (e.g., Mt. Etna^4–6^, Yasur^7^, Villarica^8^). In these cases, hazards arise from the sudden onset of volcanic activity or to fast changes of the eruptive behavior from a steady, calm state to vigorous explosive. The unexpected character of these eruptions makes them hard to predict, even at volcanoes that have up-to-date monitoring networks acquiring real-time geochemical and geophysical signals. A number of approaches have been adopted over time to forecast explosive eruptions at open conduit basaltic volcanoes, from pure geophysical^9–11^ to gas monitoring^12,13^, and many combining more than one evidence^14–18^. Despite this, the actual challenge consists in detecting the first signs of volcanic unrest in time to prevent potential disasters and fatalities.

**Figure 1:**
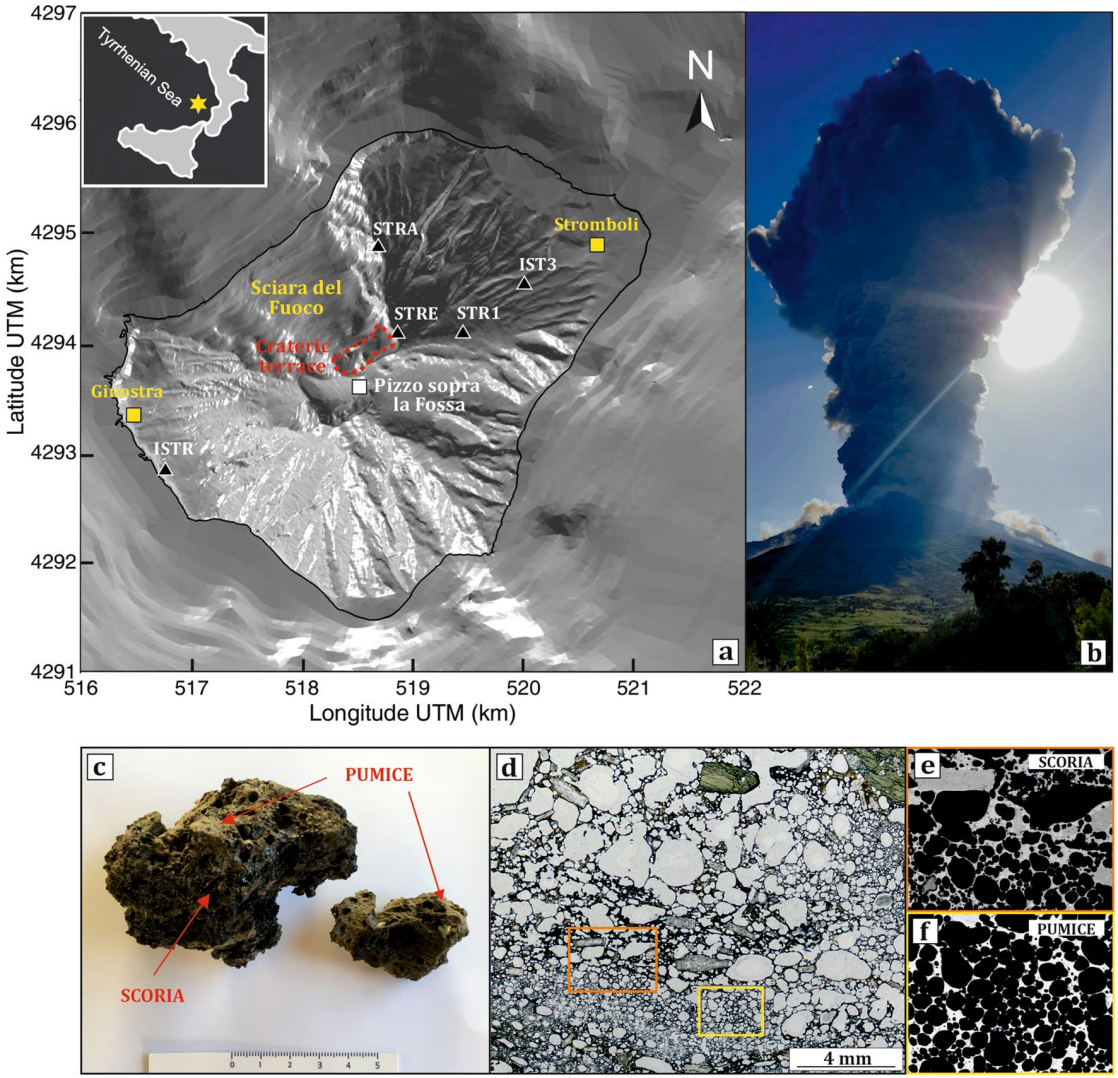
(**a**) Digital elevation model of the volcanic island of Stromboli with location of the seismic stations used in this study (triangles), the main volcanic features of the island and the two villages of Stromboli and Ginostra. The top left inset shows the position of Stromboli in the Tyrrhenian sea; (**b**) view from the village of Stromboli of the eruptive column originated at 14:46 UTC on July 3, 2019 (photo courtesy by G. Marsala); the photo shows the plume rising up to ~ 5 km of altitude at the paroxysmal eruption onset; (**c**) images of selected lapilli from the paroxysm of July 3, 2019; (**d**) optical microscope image taken under plane polarizing light for an illustrative tephra of July 3, 2019, showing the small-scale coexistence of HP [orange rectangle and blow-up in (**e**)] and LP (yellow rectangle and blow-up in (**f**)] domains within the same sample.

The paroxysmal eruptions that occurred on July 3 and August 28, 2019 at Stromboli are two exceptional examples of how the volcanic behavior at open-conduit basic systems may change drastically without apparent notice. The normal activity at the volcano consists of continuous low-energy active degassing associated to rhythmic, weak-to-moderate single explosions with ballistic ejections of pyroclastic material producing fallout over the volcano summit and the Sciara del Fuoco^19–24^ (Fig. 1a). However, the eruptive record of Stromboli is also studded by more energetic episodes with emission of blocks and bombs that can reach the coastlines and the two villages of Stromboli and Ginostra (Fig. 1a). The violent explosions at Stromboli range from small-scale paroxysms^25^ (or major explosions^26^) to large-scale paroxysms (paroxysms for simplicity^27^). Small-scale paroxysms are characterized by eruptions lasting up to 30–40 s with columns of a few hundreds of meters above the crater and ensuing dense vertically spreading plumes of pyroclastic material generally confined within 1 km. Large-scale paroxysms are the result of powerful explosions lasting from seconds to minutes, emitting pyroclastic material a few kilometers from the summit craters and producing eruptive columns 3–5 km high (Fig. 1b). Large-scale paroxysms are rare in the eruptive record of Stromboli, occurring at the scale of every 5–15 years^26,28,29^. Two other large-scale paroxysms (i.e., April 5, 2003 and March 15, 2007) are counted throughout the twenty-first century before the two episodes striking the volcanological community during the summer 2019.

The July 3 paroxysm occurred in a short sequence of two strong explosions. The first explosion was at the SW crater, while the second occurred at the central crater just ten seconds later. The explosions rapidly evolved in a ~ 5 km-high eruptive column, which in turn caused the collapse of part of the crater terrace^30,31^ (Fig. 1b). The August 28 paroxysm evolved in three main explosions. The first event, on August 28, was the most violent and produced an eruptive column of about 3 km. Two major explosions followed shortly after on August 29^31^. Both paroxysmal eruptions produced conspicuous bombs and lapilli fallout and generated small pyroclastic density currents, as well as lava flows moving along the Sciara del Fuoco. All over July and August 2019, the explosive activity at the summit craters was continuous, and remained at high level until August 31, when a general decrease in intensity started to be observed^31^.

We currently know very little about the conditions that move an open conduit volcanic system from a steady state characterized by effusive and/or weak Strombolian activity to the onset of unexpected, paroxysmal eruptions. This prevents the interpretation of geochemical and geophysical signals derived from monitoring networks. In the case of Stromboli, two divergent points of view (and relative intermediate/mixed models) have been so far proposed for explaining the source of overpressure needed to trigger an explosive event. Some authors stress the role of the injection and fast ascent of deep volatile-rich melts, the so-called low porphyric (LP) magmas, and fluids in pressurizing the shallow crystal-rich and degassed part of the conduit system occupied by the so-called high porphyric (HP) magmas^25,28,32–36^. Other authors suggest that shallow processes, such as shallow conduit clogging, shallow gas pressurization, change in magmastatic load following the extrusion of lava, determine the volcano explosivity and that the ascent of deep LP magma is a mere passive consequence of these processes^37–40^. Researchers focused on deep pressure sources emphasize the role of CO_2_ and related signals as early precursors of a paroxysm ^41,42^, whereas those focused on shallow sources stress the dominant role of fluids like the halogens^37,43^. Whatever the model, the key problem remains the identification in time of possible precursors of these violent and potentially deadly events.

Choosing the paroxysmal eruptions that occurred at Stromboli during the summer 2019 as archetypes for a multiparametric approach of investigation, this work is therefore motivated by the unsatisfactory understanding of how the timing of unpredicted, uncommonly explosive eruptions at open-conduit volcanoes can be influenced by pre-to-syn eruptive physio-chemical modifications of the magma during the final ascent. To this aim, this study highlights the high potential of in situ investigations of the erupted juvenile tephra and, in particular, of Li in plagioclase as a tracer of fluid transfer and degassing processes operating at pre- and syn-eruptive stages. Also, this study evidences how the geochemical results well integrate tilt measurements in a final model featuring the response of the system to magma plus gas movements through the conduit.

## Results

**Figure 2:**
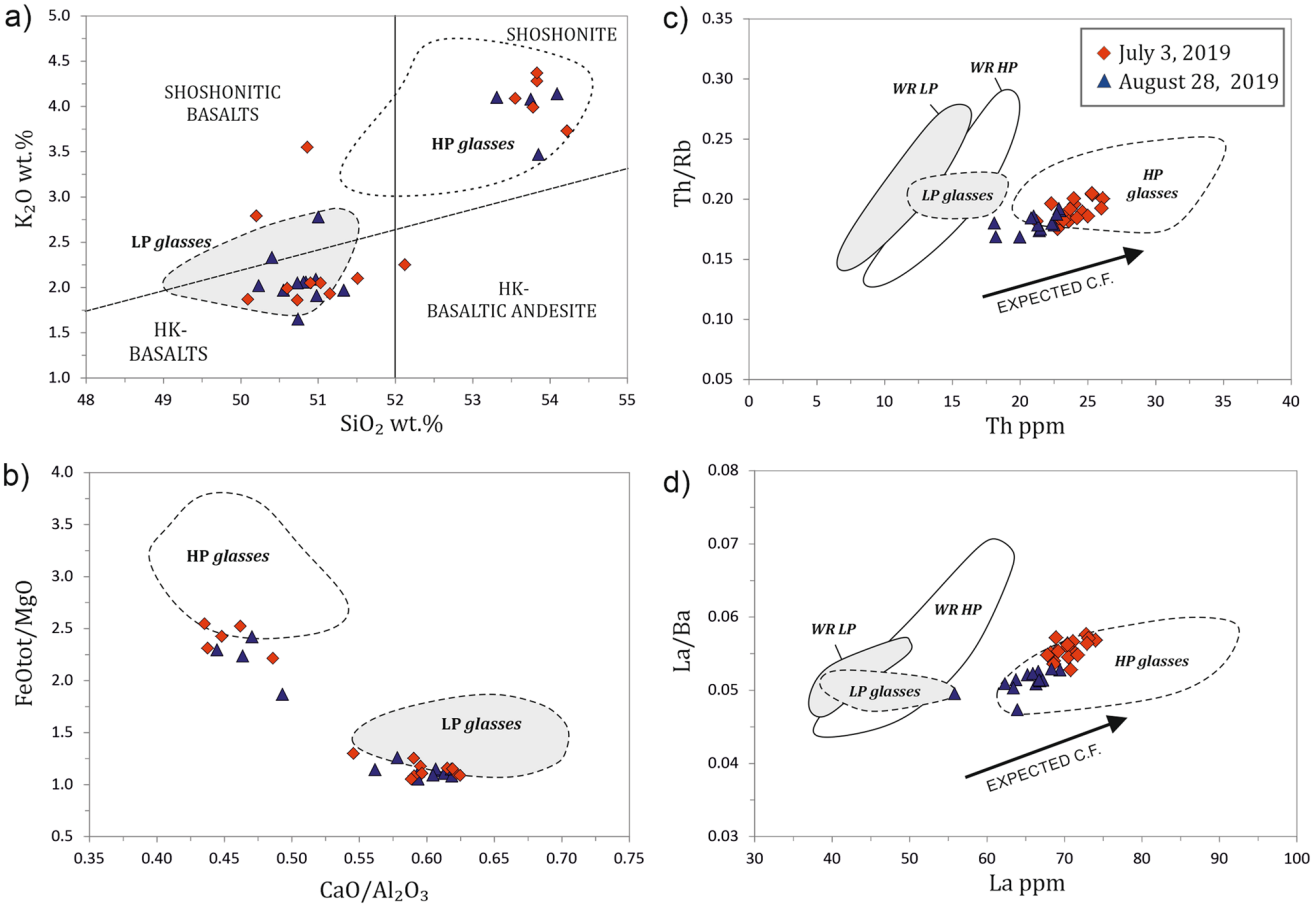
Representative diagrams for major and trace element compositions of the glassy matrix in tephra of the July 3 and August 28, 2019 eruptions: (**a**) SiO_2_ vs. K_2_O; (**b**) CaO/Al_2_O_3_ vs. FeO_tot_/MgO; (**c**) Th vs. Th/Rb; (**d**) La vs. La/Ba. In diagrams (**a**) and (**b**), the 2019 glass compositions are shown together with compositional fields for HP (white) and LP (light gray) glasses emitted during large scale paroxysms since the fifteenth century. Data are from Refs.^29,33,36,90^, and refer to anhydrous values. In diagrams (**c**) and (**d**), the July 3 and August 28, 2019 glass compositions are plotted together with the compositional fields for both HP and LP glasses (dashed lines) and whole rocks (WR; solid lines). HP and LP fields refer to compositions of pumice and scoria of paroxysmal eruptions from 1998 to 2005 (data from Refs.^29,44,62,92^). C.F.: crystal fractionation.

According to previous petrological studies and field observations^28,29,32–36^, large-scale paroxysms at Stromboli are characterized by the emission of a nearly degassed and highly-porphyric (HP) magma residing in the volcanic conduit at shallow levels (2–4 km below the summit) that interacts with gas-rich, low-porphyric (LP) magma coming from the deep levels of the volcano plumbing system (7–10 km below the summit). During paroxysmal eruptions, the deep-seated LP magma can rise under dominant closed-system conditions emitting highly vesicular basaltic pumices in addition to a shoshonitic crystal-rich scoria that is typically ejected during the ordinary activity^28,29,32,36^. Even tephra emitted during the July 3 and August 28, 2019 paroxysms are shoshonitic to high-K basaltic juvenile fragments with clear evidence of interaction between HP and LP magmas (Fig. 2). We analyzed lapilli and bombs (1–10 cm in size) from both paroxysmal eruptions (Fig. 1c). Lapilli and bombs were collected at different sites 2.0–2.5 km far from the crater terrace on the western part of the island. The sampling was performed during the eruption itself or shortly after the end of eruption. All samples show textures characterized by evident pumice/scoria pairs (Fig. 1c,d). The scoria portion is dark, poorly vesicular (10–30 vol% vesicles as determined by optical microscope inspection) and rich in crystals (Fig. 1d,e). It contains 45–50 vol% of plagioclase, clinopyroxene, and olivine set in a glassy matrix of shoshonitic composition (53–54 wt% SiO_2_; Fig. 2 and Supplementary Dataset File 1). The pumice portion is light in color, strongly vesicular (50–70 vol% vesicles) and crystal poor (Fig. 1d,f). It contains 5–10 vol% of the same crystals set in a basaltic glassy matrix, with higher abundances of olivine and clinopyroxene with respect to plagioclase. Dark scoria and light pumice are strictly intertwined in the same ejecta, so that the separation of one portion from the other was not possible (Fig. 1c,d). This interaction is evident from the composition of the matrix glass of any individual sample, where we found both the HP and LP magma compositions, plus some intermediate values between the two end-members (Fig. 2). The trace element variability of the matrix glasses also emphasizes the greatest geochemical affinity of the analyzed products with the composition of the HP magmas, with the July 3 glass compositions that are, overall, slightly more evolved than those of August 28, 2019 (Fig. 2 and Supplementary Dataset File 1).

**Figure 3:**
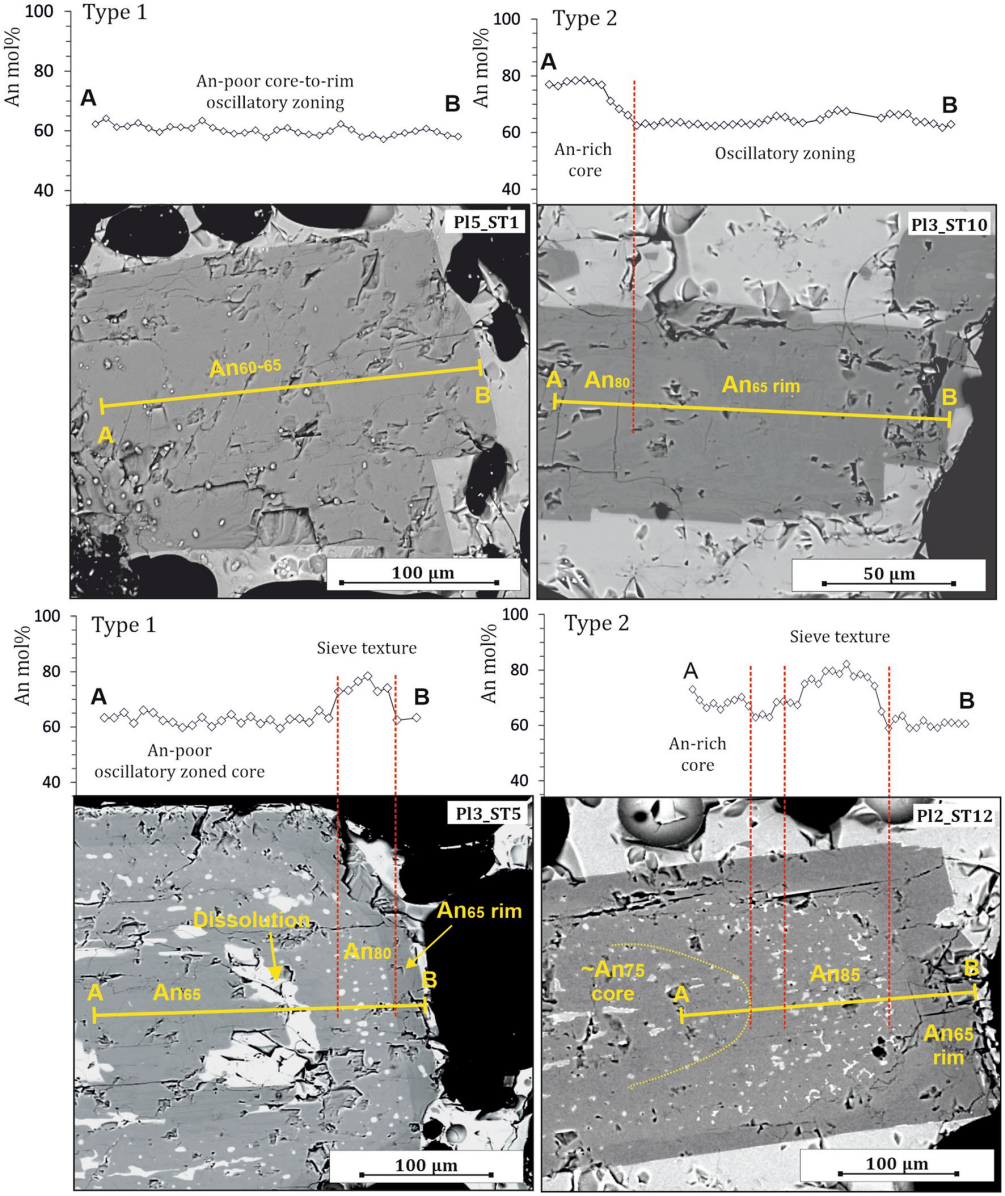
Core-to-rim An mol% profiles, coupled with BSE images of plagioclase crystals representing the two dominant zoning types, i.e. Type 1 (low-An core composition) and Type 2 (high-An core), defined in this study. Yellow straight solid lines, indicated as A–B, mark the analyzed profile of the crystal.

Plagioclase is the dominant mineral phase in each clast (~ 50 vol% of crystals) and occurs as zoned crystals with size of 250–2000 µm measured along the longest axis. Plagioclase is affected by a variety of disequilibrium textures and dissolution surfaces from core to rim. Rarely, it occurs as clear oscillatory-zoned crystals without any evidence of dissolution/resorption textures. Two types of plagioclase have been recognized from the examination of the major element zoning of 78 crystals (49 crystals from the paroxysm of July 3, and 29 crystals from the August 28 paroxysm) dominantly dispersed within the scoriaceous portion of each clast (Fig. 3; see also Supplementary Dataset File 2). A dominant type, hereafter Type 1, has oscillatory zoned profiles characterized by low amplitude An oscillations, varying on average between An_55_ and An_65_ (Fig. 3). Type 1 has euhedral to partially dissolved inner cores, but no significant An variation between inner cores and rims, except in presence of sieve textures where the An content sometimes increases up to ~ 86 mol%. The second type, hereafter Type 2, has euhedral to anhedral inner cores at high An content, ranging from An_75_ to An_85_ (Fig. 3). The An-rich cores are overgrown by euhedral rims with composition identical to those of Type 1 (i.e., An_55–65_). Moreover, rims may record or not increasing An concentration in correspondence of sieve textures, as for crystals of Type 1. Noteworthy, the two zoning types distribute with equal percentage in both deposits of July 3 and August 28, 2019, that is of 71–72% for Type 1 and 28–29% for Type 2. The range of plagioclase compositions, together with the close association of scoria and pumice in the same clast, supports the assumption that both paroxysmal eruptions were the products of syn-eruptive mingling of two magmas characterized by distinct physio-chemical conditions^28–44^.

Compositional variations of FeO and some trace elements (Mg, Sr, Ba, Sr/Ba, Li) have been compared with An compositions on a selection of 19 crystals (i.e., 11 crystals from the paroxysm of July 3, and 8 crystals from the paroxysm of August 28; Supplementary Dataset File 2 and 3). Among the others, these crystals were chosen for the poor evidence of resorption and/or fracturing at their rims that could alter the analytical reliability, and therefore hide the record of shallow processes potentially affecting the final stage of crystal growth. The FeO content is typically in the range 0.6–1.2 wt%, while Mg varies between ~ 700 and ~ 1200 ppm. Concentrations of both elements remain rather constant in the An-rich cores of Type 2 plagioclases, even when resorption textures (e.g. coarse sieve textures) occur. FeO and Mg increase up to ~ 1.5 wt% and 2200 ppm respectively in sieve textures observed at the plagioclase outer core and/or rim. In all crystals, Sr/Ba ratios vary within a narrow range of 1.2–3.3, with values that are on average more constant (~ 2.5 to 3) in Type 1 oscillatory zoned crystals. The Sr/Ba fluctuations correlate well with variation in An content across the plagioclase, showing values in the calcic inner cores that are usually higher than those measured toward the rim. The Sr ppm abundance also closely follows the An content in the majority of cases. Other element trends in our analyses were not examined to the extent of An, Fe, Mg and Sr/Ba, but display increasing chemical trends toward the edge of plagioclase.

**Figure 4:**
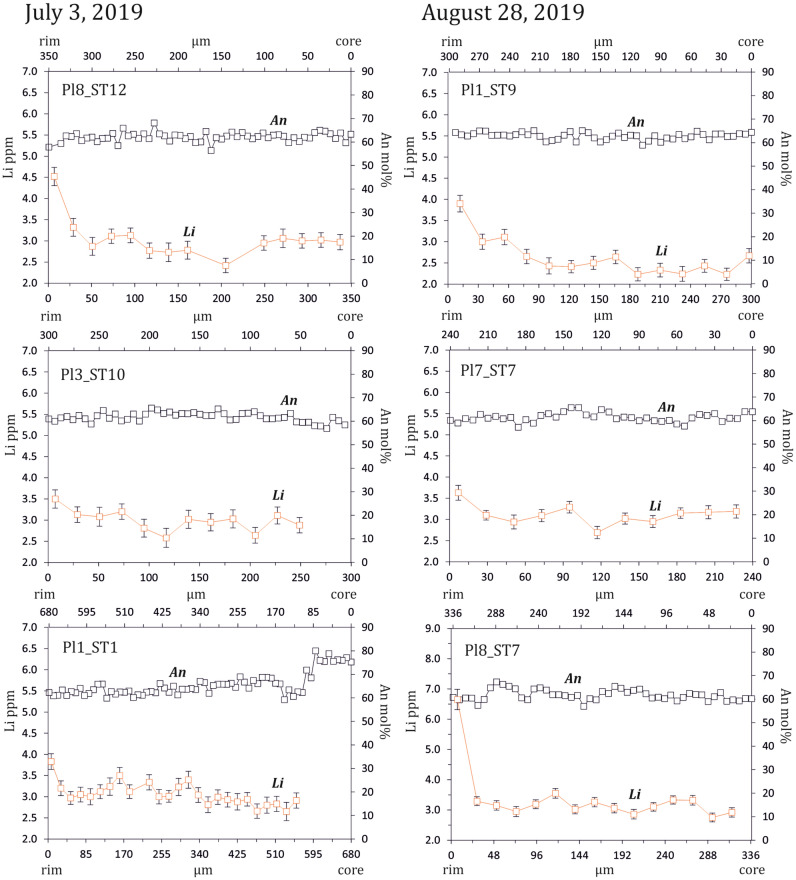
Compositional traverses for Li (ppm) and An (mol%) of representative plagioclase crystals. Error bars are the standard deviation of the mean of measurements (± 1σ). Error bars for the An content lie within the square symbol and are not shown.

We focused on the relative abundance of Li and other mobile elements, such as alkali metals (K_2_O and Na_2_O wt%), as well as other elements with similar plagioclase-melt partitioning (e.g. Ba), because they may offer insight into processes of fluid transfer and degassing operating before and during the eruption. In particular, the rapid diffusion of Li at shallower depths allows it to capture processes occurring in volcanic systems at pre- or even syn-eruptive stages^45–49^. The concentration of Li in plagioclase is in the range 2.2–6.7 (± 0.3–0.7) ppm (Fig. 4 and Supplementary Dataset File 3). Analytical traverses from the outer core of plagioclase display rather constant or oscillatory variation of Li followed by an increase at the rim. The Li increase approximately occurs across the 50–90 µm-wide outer rim of any plagioclase, with variations up to 6.7 ppm Li. Noteworthy, the individual crystals having increasing Li trends also show limited compositional variability in An along the same portion of the rim enriched in Li (Fig. 4 and Supplementary Dataset File 3). Apart from the plagioclase outer rim, Li is considerably enriched in the glass portion close to the crystal edge (13–17 ± 0.4–1.4 ppm). The variability observed for Li was not detected for other compatible trace elements, such as Sr, whereas other divalent cations (Mg, Ba) and LREE weakly correlate with Li in few crystals. Instead, a good correlation occurs with respect to K_2_O and Na_2_O wt%, which show similar increasing trends rimward.

**Figure 5:**
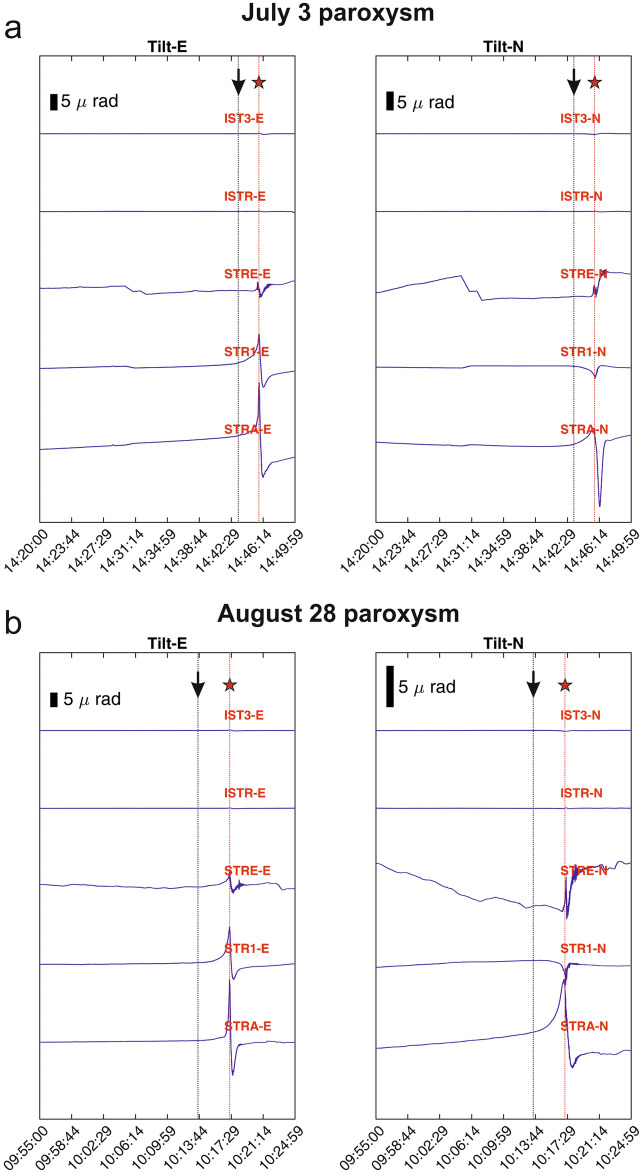
Tilt signals accompanying the July 3 and August 28, 2019 paroxysms. The red stars indicate the time of the paroxysms, and the arrows indicate the onset times of the inflation as detected by STA/LTA algorithm.

**Figure 6:**
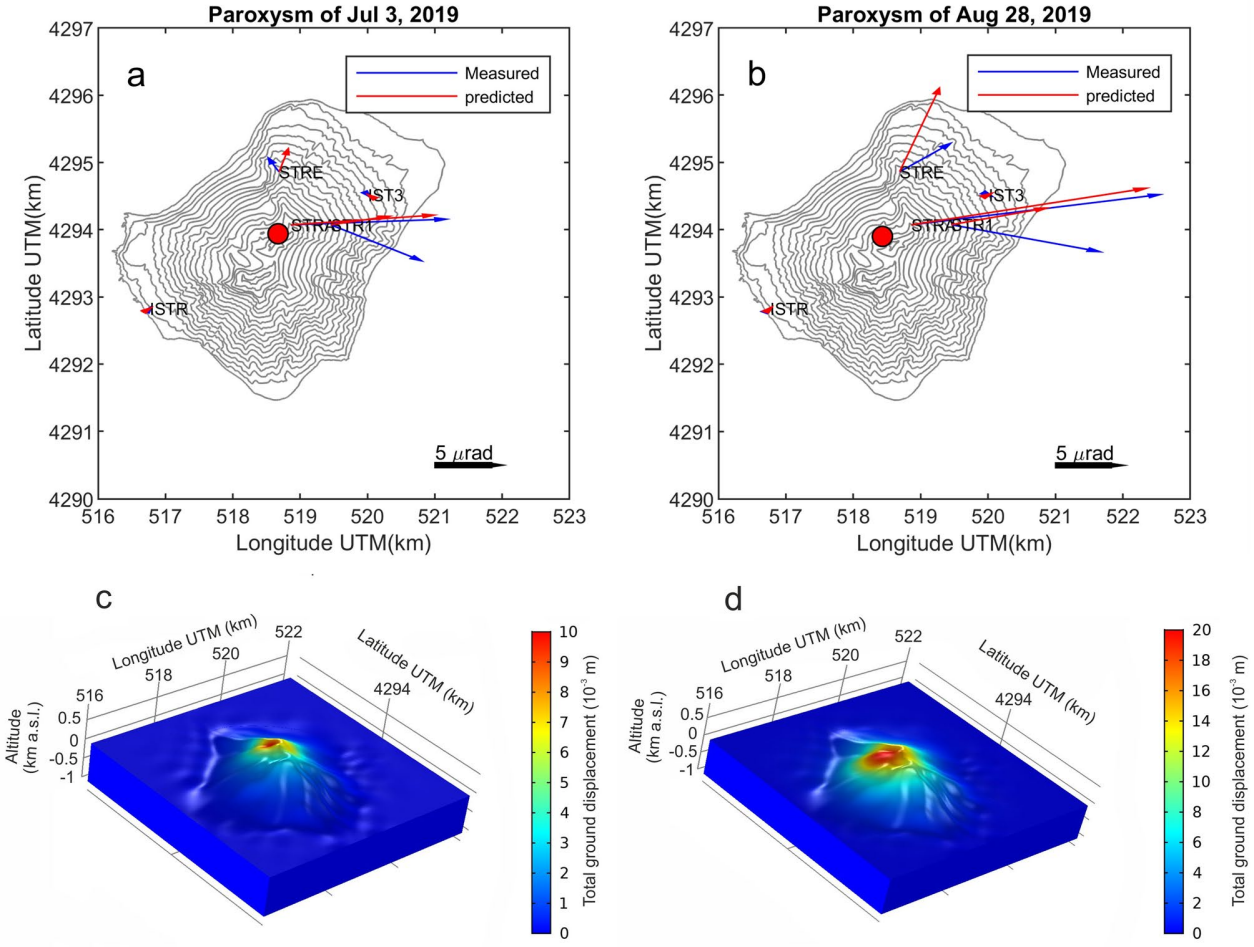
(**a**,**b**) Digital elevation model of Stromboli showing the comparison between measured (blue arrows) and predicted (red arrows) tilt vectors during the July 3 and the August 28 paroxysms, respectively. Downward dipping directions are shown by arrows. The red dots indicate the location of the modelled pressure sources. (**c**,**d**) Spatial distribution of the maximum total ground displacement during the July 3 and the August 28 paroxysms, respectively.

Petrological data have been integrated with tilt time series accompanying the two paroxysmal eruptions, which have been derived by means of signals recorded by 5 broadband seismometers (Fig. 1a). Seismograms related to the paroxysmal eruptions taking place at 14:46 GMT of July 3 and at 10:17 GMT of August 28 show the occurrence of strong explosion-quakes (see Supplementary Information). The tilt time series clearly show stronger tilt changes at the stations located at higher altitude, with maximum tilt values at STRA located at 842 m asl, equal to 11 and 18 μrad along the East–West component, recorded during the July 3 and the August 28, 2019 paroxysms respectively (see “Methods”, Table 1 and Figs. 5, 6). These data document a volcano inflation pattern started 2.4–3.4 min before the July 3 and the August 28 paroxysms, respectively, with stronger inflation accompanying the second one (Table 1).

**Table 1:** Results from finite element (FE) modeling for the two paroxysms.

Station	Coordinates UTM			3 July 2019						28 August 2019					
				Tilt x (μrad)			Tilt y (μrad)			Tilt x (μrad)			Tilt y (μrad)		
	Easting (km)	Northing (km)	Altitude (m)	Measured	Predicted	σ	Measured	Predicted	σ	Measured	Predicted	σ	Measured	Predicted	σ
STRA	4294.07	518.86	842	− 11.4892	− 10.571	1.84	− 0.41933	− 0.718	2.42	− 18.3927	− 17.368	1.34	− 2.2419	− 2.707	2.29
STR1	4294.07	519.45	561	− 6.7159	− 4.329	1.5	2.6747	− 0.619	0.34	− 11.0229	− 7.081	2.82	1.9959	− 1.235	0.4
STRE	4294.85	518.68	436	0.82778	− 0.691	0.36	− 1.0449	− 1.73	0.72	− 3.7841	− 2.956	1.02	− 2.1053	− 6.217	1.41
ISTR	4292.82	516.76	70	− 0.1205	− 0.116	0.03	− 0.13344	− 0.0636	0.02	− 0.10494	− 0.156	0.12	− 0.07179	− 0.124	0.04
IST3	4294.51	520	255	− 0.17033	0.0272	0.03	0.15717	0.0148	0.02	− 0.25886	− 0.238	0.19	0.11069	− 0.102	0.1
Spherical model parameters				Estimated			σ			Estimated			σ		
UTM Easting (km)				518.67			93.38			518.43			175.27		
UTM Northing (km)				4293.94			54.77			4293.9			70.71		
Altitude (m)				326.314			42.44			165.1			77.68		
Cavity volume change (10^3^ m^3^)				6.5			0.6			30			3		

The upper part reports the measured tilt components for the considered stations together with those predicted by the models and their calculated uncertainties (σ). The estimated parameters of a spherical source model and their uncertainties are reported at the bottom of the table. The models were obtained by maximizing the fitting between predicted and measured tilts using a Monte Carlo approach.

## Discussion

We emphasize that Li diffusion in plagioclase is rapid enough to provide temporal constraints on short-lived processes. It is widely assumed that quenching of juvenile clasts during fragmentation effectively locks in the signal preserved in volatile elements such as Li^48,50–53^. Thus, the distribution of Li in plagioclase rims can reflect changing conditions during the eruption itself. Understanding the mechanism responsible for the Li increase requires, at first, a clear definition of the magmatic processes occurring just before or upon eruption. A key to understanding the origin of the high Li content of the plagioclase rims is determining whether increasing Li profiles in plagioclase reflect variations in plagioclase-melt partitioning. Main controlling parameters on plagioclase-melt partitioning are the An content and the temperature variations^54,55^. We can exclude the effects of the former because the anorthite composition across the rims of plagioclase phenocrysts is almost identical, and there is no correlation between An and Li across the same zones where Li increases. This rules out any potential control on Li partitioning based on An variations. Temperature changes could play a more important role than An content on Li partitioning into plagioclase. The numerical model of Ref.^56^ at non-isothermal conditions predicted a range of magmatic temperatures of 1077–1112 °C in the upper conduit of Stromboli (from a depth of ~ 1000 m up to the vent), with temperatures that become higher close to the vent. Increasing temperatures should make Li more compatible in plagioclase^54^, thus promoting the ingress of Li into the crystal. However, the influence of temperature on plagioclase-melt partitioning cannot explain the elevated Li contents at the edge of plagioclase phenocrysts, because at high magmatic temperature (> 1100 °C) even a temperature increase of around 30 °C would produce insignificant variations of the Li partition coefficient^54^, which are anyway within the analytical error of K_D_ determinations (i.e. ~ 0.002^54,57^). The melt composition can also have a role in controlling Li abundances in plagioclase, because Li is moderately incompatible in plagioclase (^plg/melt^K_D_ = 0.1–0.7^54,57,58^) and hence, continued crystal growth would increase the Li contents of the melt. This enhances the partition of Li into plagioclase, but only under the assumption that the melt is not depleted in Li during degassing^53^. As Li is extremely mobile and preferentially partitions into the gas phase relative to the melt^59^, the interpretation of Li enrichments cannot rely on simple plagioclase/melt equilibrium partitioning changes, but it requires an alternative explanation. Gas flushing from deep magmas can transfer high Li concentrations to shallow levels, but because Li diffuses more rapidly than all other trace elements in the melt^60^ and in plagioclase crystals^61^, Li will be lost due to gas phase separation in both phases on very rapid timescales (i.e., hours, minutes or seconds depending on magmatic temperatures and compositions). Indeed, the low Li contents recorded along the entire profile of plagioclases of the Stromboli paroxysms, except at their outer rims, indicate that the magma has lost volatiles before its final ascent. This agrees with prolonged plagioclase crystallization and convection/recycling within the HP degassed magma^33,44,62–64^. As plagioclase grows within the HP magma, gas fluxes from the deeper undegassed reservoir may cyclically carry on and accumulate Li in the shallow HP system. The addition of Li in the melt due to gas flushing has been invoked by Ref.^49^ to explain the presence of Li enrichments in plagioclase of the recent activity of Mt. Etna. This mechanism could also justify the increasing Li concentration toward the plagioclase rim at Stromboli, through a process of equilibrium concentration between the Li-poor plagioclase and Li-rich melt. However, this process fails to explain the preservation of high Li concentrations at the plagioclase edges during the final stage of magma ascent and fragmentation, since the HP magmatic system would be already degassed (totally or just partially) before being decompressed. Long-lasting magma recharge and mixing with a Li-rich LP melt can be also excluded, because no other elements indicating changing melt concentration (i.e. Mg) correlate with the increase in Li toward the plagioclase rims. To explain the preservation of the high Li content at the plagioclase edge, the attention needs to be turned to the complexity of shallow conduit processes, invoking mechanisms of Li transport into the gas phase and subsequent transfer back from the gas to the melt, and then into plagioclase. The mechanism responsible for the transfer of Li from the gas phase to the melt still is matter of debate, but it has been previously related to the upward migration of an exsolved gas phase rich in alkali and fluid mobile elements^45,50,65^, along with the capability of Li to form chlorine complexes in melts and fluids during decompression^45,48^. Existing models, originated from experimental studies^59^, demonstrated that in silicic magmas Li and other alkalis preferentially partition into an aqueous fluid phase over melt, especially at low pressure (~ 50 MPa), and the presence of significant chlorine further promotes Li partitioning. The fluid-melt partition coefficient of Li (as such other volatile and soluble elements) increases with decreasing pressure, so that Li concentrations greatly increase in the gas phase during migration to shallow levels. Further decompression of the system at lower P_H2O_ allows the fluid to reach a threshold pressure where the fluid separates into a low-density vapor coexisting with a dense brine rich in alkali metals, which is able to re-equilibrate with the surrounding melt^65^. The extraction of significant amounts of chlorine (in the form of HCl) into the vapor phase during this process has important implications on Li partitioning, because Li forms hydroxides in absence of chlorine under subcritical fluid conditions^66^. The reaction makes Li much less compatible in fluids and melts, enhancing the actual migration of Li back into the plagioclase. The presence of high Cl contents in primitive melt inclusions from both HP and LP magmas at Stromboli (up to 2900 ppm^33,44^), and the correlation that we observed in plagioclase between Li and alkalis supports the argument detailed here, though the faster diffusion of Li would lead Li to migrate between brine, melt, and plagioclase at rates faster than other elements. We stress here that the process of gas accumulation and re-incorporation of Li must have happened at very low pressure and have been directly associated with the onset of the eruption, otherwise the Li enrichments would have been fully re-equilibrated. Our assertion also originates from Li diffusion modeling results, showing that the enrichment of Li at the rim of plagioclase formed in 2–180 (± 1–30) seconds before the eruption (see Methods and Supplementary Dataset File 4). This deduction is also supported by the inversion of the calculated tilt variations, which constrains the pressure source location for both the paroxysmal eruptions at very shallow levels, i.e. within two hundred meters above the sea level (Fig. 6; see Methods). Our model reproduces sources of pressurization that are slightly below the typical source locations of the very long period (VLP) events at Stromboli^67–69^. Considering a Young's modulus of 75 GPa, the source volumes associated with these variations were estimated to be on the order of 6.5 × 10^3^ m^3^ for the July 3 and 30 × 10^3^ m^3^ for the August 28, 2019 paroxysms. Previous estimations of volume changes accompanying “normal” Strombolian activity at Stromboli, performed by moment-tensor inversions of VLP events, provided values on the order of 10^2^ m^3^^70,71^. Hence, it appears that the 2019 paroxysms were triggered by the uprise of a much larger volume of magma plus gas with respect to the typical Strombolian activity.

**Figure 7:**
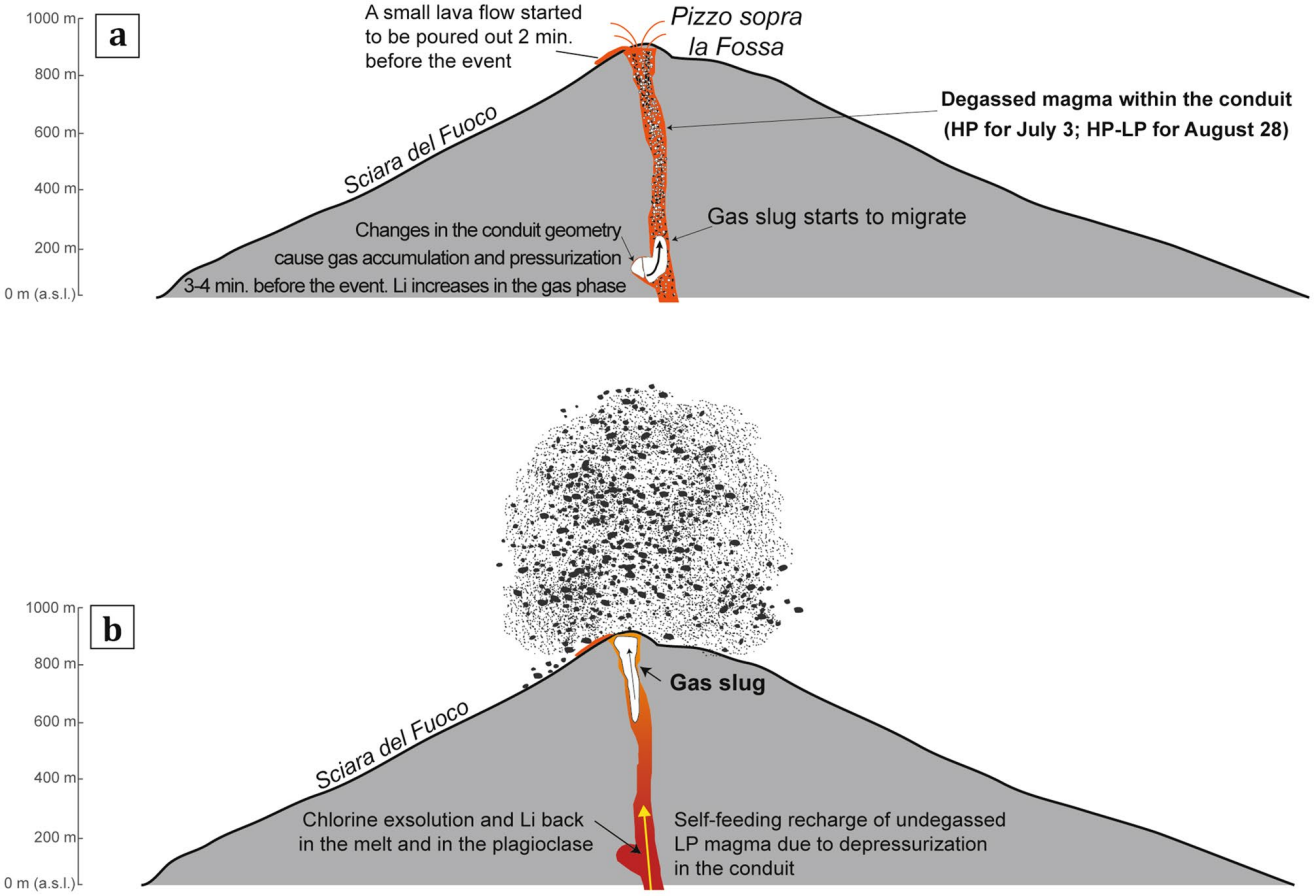
Interpretative model of the plumbing system dynamics at Stromboli leading to the large-scale paroxysms of July 3 and August 28, 2019. Panels (**a**) and (**b**) refer to subsequent magmatic events within the upper conduit. (**a**) Continuous gas replenishment pressurizes the upper volcanic conduit at around 200 m a.s.l., yielding to an incipient gas slug. As the gas slug grows and moves upwards to the surface, it pushes up the overlying column of degassed HP magma. Decompression driven degassing promotes the loss of Li from the melt, while increases its concentration in the gas slug. (**b**) As the system is further decompressed, the fluid separates into a hydro-saline liquid (brine) and a supercritical vapor, thus creating proper conditions for partitioning of Li from the gas slug back to the melt. The pressure release within the conduit triggers a mechanism of self-feeding magma recharge, with recalling of the undegassed LP magma from depth.

Unquestionably, the tilt time series record an inflation of the magmatic source in the upper conduit that started 2.4–3.4 min before both eruptions. Determinations coming from the Li diffusion in plagioclase and the tilt time series give the opportunity to reconstruct the escalation of phenomena leading to the paroxysmal eruptions at Stromboli during the summer 2019. Our data imply that the gas recharge/discharge rate could have been interrupted at very shallow levels (i.e., two hundred meters above sea level), probably as a consequence of an interruption of the gas flux in the shallow conduit that occurred a few minutes before the eruption (Change in the conduit geometry? Transient obstruction? Fig. 7a). Indeed, gas flux disruptions are not a novelty in the eruptive history of Stromboli, as the presence of flow disruption sites in the shallow plumbing system (from the sea level to a few hundred meters below the craters) has been inferred in the past by VLP waveform inversions^69–71^. Development of the ensuing pressurization was therefore due to the continuous gas replenishment characterizing the ordinary degassing activity of Stromboli. The first effect of the growing pressure was the formation of a gas slug in the modified conduit (Fig. 7a), which triggered the piston-like upward migration of the HP magma occupying the last 400–500 m of the conduit. This finally produced a modest lava effusion two minutes before the paroxysmal eruption of July 3 (http://ingvvulcani.com; Fig. 7a). The pressure release accompanying the upward migration of the gas slug and the lava effusion at the surface triggered a self-feeding recharge of the deeper, undegassed LP magma, whose fast ascent caused decompression-driven degassing coupled with massive chlorine exsolution (Fig. 7b). The whole process leading to change in the partitioning behavior of Li between melt, gas and plagioclase occurred in less than 180 s before the astonishing explosion of July 3 (Fig. 7b). In the weeks preceding the August 28 paroxysm, the activity at the summit craters of Stromboli was rather intense (VLP > 22 events per hour; high volcanic tremor; data from the LGS at http://lgs.geo.unifi.it), which is consistent with the presence of undegassed LP magma in the conduit (Fig. 7a). Dynamics leading to the escalation of volcanic phenomena on August 28 appear rather similar to those of July 3, suggesting that the transitory conditions allowing pressurization in the shallow conduit were not restored after the July 3 eruption (Fig. 7b). On the contrary, the end of the August 28 paroxysm marked a prominent drop to green levels for all the monitoring parameters (VLP, infrasound, volcanic tremor; data from the LGS at http://lgs.geo.unifi.it) and the return to the ordinary volcanic activity, hence indicating a complete re-establishment of the gas flux recharge/discharge ratio in the volcanic conduit.

Our model suggests that the escalation of volcanic phenomena during the summer 2019 at Stromboli started from an accidental event in the very shallow conduit of the volcano. The ensuing rapid changes of degassing conditions at superficial levels limited the development of mid-to-long term precursory signals, emphasizing therefore the rather unpredictable character of such kind of eruptions at present, and the crucial role played by the gas phase in the sudden activation of vigorous explosive activity from an ordinary, steady state. The possibility of such rapid (< 5 min) shifts from weak to high explosivity at Stromboli underscores the need for improving the existing monitoring networks with early warning systems capable to detect further very short-term signals that promptly anticipate an incoming paroxysm, minimizing the risk.

## Methods

### Microanalytical procedures

Plagioclase crystals from twenty-three pumice and scoria fragments were analyzed in polished thin sections. Textures and major element zoning were studied using a combination of back-scattered electron images (BSE; 1024 × 864 pixels) and scanning electron microscopy at the Dipartimento di Scienze Biologiche, Geologiche e Ambientali, at the University of Catania, Italy. Major element compositions of plagioclase were measured by a Tescan Vega-LMU scanning electron microscope (SEM) equipped with an EDAX Neptune XM4–60 microanalyzer operating by energy dispersive system (EDS). The microscope has an ultra-thin Be window coupled with an EDAX WDS LEXS (wavelength dispersive low-energy X-ray spectrometer) that is calibrated for light elements. Instrumentation and operating conditions were set at 20 kV accelerating voltage with 8 nA and 2 nA beam current to obtain respectively high-contrast BSE images and the analysis of major element abundances. Repeated analyses on internationally certified An-rich plagioclase and glass standards during the analytical runs ensured precision of around 3–5% for all elements, while accuracy is ~ 5%. The major element abundances were measured along core-to-rim traverses in 78 plagioclase crystals. Analytical traverses were done parallel to the longest side of plagioclase with spacing between individual analytical spots of 5–9 µm.

Trace element analyses were acquired by laser ablation inductively coupled plasma–mass spectrometry (LA-ICP-MS) at the Dipartimento di Fisica e Geologia, University of Perugia (Italy). In detail, we used a Teledyne Photon Machine G2 laser ablation system connected to a Thermo Fisher Scientific iCAP-Q ICP-MS^72,73^. Helium was utilized as carrier gas with Ar and N_2_ added just after the ablation cell to avoid plasma destabilization and heighten the instrumental sensitivity, respectively. Before each analytical session, the LA-ICP-MS operating conditions were optimized by continuous ablation of NIST SRM 612^74^ glass reference material to provide maximum signal intensity and stability for the ions of interest and reducing potential interferences. The ThO^+^/Th^+^ ratio was used as a proxy for oxide production and maintained below 0.5%. The sensitivity and the stability of the system were then evaluated on ^7^Li, ^139^La, ^208^Pb, ^232^Th, and ^238^U by a short-term stability test. It consisted of 5 acquisitions (one minute each) on a linear scan of NIST SRM 612 glass reference material.

Measurements were done on a total of 19 plagioclase crystals using a 12 × 50 μm rectangular laser beam oriented with the major axes parallel to the shortest side of the crystal. The separation between individual spot analyses was 8 μm. The laser fluence and repetition rated was ~ 4 Jcm^−2^ and 10 Hz, respectively. For all analyses, we acquired 25 s of background signal, followed by 40 s of ablation signal, and 25 s of washout. Data Reduction was carried out following the procedure reported by Ref.^75^, and using the Iolite v.3 software package^76^. The NIST SRM 610^74^ and the USGS BCR2G^77^ reference materials were used as the calibration standard and quality control, respectively. Si concentration determined by SEM–EDS/WDS was used as the internal standard. The reference materials were analyzed at the beginning and the end of each plagioclase transects to monitor and correct instrumental drifts. Under the reported analytical conditions, precision and accuracy are typically better than 10%^72,73^. Repeated analyses of the USGS BCR2G^77^ resulted in an average value of 9.7 ± 0.26, corresponding to a 1-sigma precision and a deviation from the reference values of 2.6% and 8%, respectively. The repeated analyses of the USGS BCR2G are reported in the Supplementary Dataset File 3. The relatively large size of the LA-ICP-MS beam implied the possibility of contamination of the analyses in plagioclase by the ablation of the hosting glass^78^, making necessary to discard some analyses collected at the plagioclase edge. Each plagioclase time resolved signal was checked for the increase in concentrations of selected incompatible trace elements, potentially indicating glass contamination. Non-compliant analyses were rejected before modeling.

### Diffusion modeling

Li diffusion modeling was carried out in one dimension using a modeling approach in which the diffusion equation was solved analytically^79^. We have used a uniform initial Li concentration (C_0_) within the plagioclase and a constant concentration at the plagioclase-melt interface (Cs) as the initial and boundary conditions for the error function (*erf*):

$$\left[ {\left( {{\text{Cx}} - {\text{C}}_{0} } \right)/\left( {{\text{Cs}} - {\text{C}}_{0} } \right)} \right] = 1 - erf\left[ {{\text{x}}/2\left( {{\text{D}}t} \right)^{0.5} } \right]$$

Cx is the concentration at the distance x from the crystal-melt surface; *t* is the time since diffusion begins; D is the diffusion coefficient for Li in plagioclase (Supplementary Dataset File 4). The melt is assumed to have acted as a semi-infinite reservoir of Li, with diffusion being the cause of Li enrichments at the plagioclase rim. Hence, as boundary concentrations (Cs), we have used the Li content measured at the edge of any crystal, which was held constant during modeling. For the initial Li distribution (C_0_), we have considered the lowest Li concentration across the 100–150 µm-wide inner rim of any single crystal. Li diffusion in plagioclase is assumed to be independent from plagioclase An content, which is consistent with experimental data available^61,80^. We have used two different An-independent diffusion coefficients of 1.09 × 10^−10^ m^2^/s and 6.78 × 10^−12^ m^2^/s on the basis of data provided by Refs.^61^ and ^80^ respectively, in order to explore the whole range of possible diffusion timescales at the investigated temperatures. D_Li_ values were extrapolated at temperatures of 1100 °C, that is slightly higher than those experimentally calibrated (up to 1050 °C^80^). The chosen temperature accounts for the variation in temperature within the upper (to the depth of 1000 m) conduit of Stromboli^56^. Extrapolation of diffusion coefficient to different temperatures than those experimentally calibrated is not uncommon in studies that use diffusion chronometry to model natural processes^81,82^. Here, extrapolation to 1100 °C is feasible given the linear dependence of logD_Li_ on temperature^61,80^. Moreover, variations of temperatures within a range of around 300 °C do not produce significant changes in the activation energy for Li diffusion in plagioclase, which would imply a change in the diffusion mechanism^61^. Using the diffusion coefficient based on Ref.^61^, Li profiles can be matched for diffusion occurring over just 2–15 s, whereas the diffusion coefficient provided by Ref.^80^ gives a range of timescales between 16 and 180 s (Supplementary Dataset File 4). Temperatures are the largest source of uncertainty in constraining proper values for D_Li_ and in the modeled diffusion timescales. However, the calculated timescales are minima as the modeling is carried out by using pre-eruptive temperatures that are the highest estimates at Stromboli, and account for changes in crystallinity, gas discharge and rheological properties of the system during an eruption^56^. We considered a temperature uncertainty of ± 30 °C, which when propagating into the time estimates leads to relative errors of 17–55%. The adoption of an analytical solution for the diffusion equation also produces diffusion timescales that are underestimated, mostly because of the non-dependence of Li diffusivity from composition or other thermodynamic parameters except temperature. However, without further constraints on the cooling and ascent history upon eruption, more detailed modeling would be not justified.

### Geophysical signals

The seismic signals used to derive tilt data were recorded by 5 broadband seismometers equipped with Guralp CMG40T (STRA, STR1 and STRE) and Nanometrics Trillium 120 s (ISTR and IST3), belonging to the monitoring seismic permanent network run by Istituto Nazionale di Geofisica e Vulcanologia (Fig. 1). To turn the seismic signals into tilt, we followed the method proposed by Ref.^83^, whose main steps consisted of: (1) filtering the seismic signal by a causal low-pass filter below the natural frequency of the sensor, (2) integrating it; and (3) multiplying the result by—*S ω*_0_^2^/g, where *S* is the seismometer sensitivity, *g* is the gravitational acceleration, and *ω*_0_ is the natural angular frequency. A similar method was also applied at Stromboli volcano and highlighted persistent inflation-deflation cycles, associated with the continuous Strombolian activity^84^.

To detect the onset of the inflationary patterns, the two tilt time series of STRA station, chosen according to the highest tilt values, were taken into account. The STA/LTA (Short Time Average/Long Time Average^85^) algorithm, routinely used in seismology to pick the phases of earthquakes, was applied on such tilt time series with the following parameters: short time window of 60 s, long time window of 600 s and STA/LTA ratio of 2.2. The inflation onset times, detected by this method, preceded the first and second paroxysms by 2.4 and 3.4 min, respectively. The uncertainty of these temporal estimations, equals to ± 30 s, depends on the short window used in the detection, and is calculated as half of its duration.

To constrain the location of the pressure source and model it, we adopted a finite spherical body^86^ with 5 unknowns (3 coordinates, radius, and pressure variation). Firstly, we considered the source buried into a linear, homogenous and isotropic half-space. Nonlinear inversions were performed by using a combination of trust-region-reflective least squares^87^ and mesh adaptive search^88^ algorithms. We considered a varying depth model^89^ to account for volcano topography. Results indicated a source within the volcanic edifice, above the two lowest stations, thus violating the half-space assumption of the model. Consequently, we abandoned the half-space assumption and inverted the tilt data using a numerical approach. In particular, we employed a finite element (FE) modeling that accounts for the prominent topography and the extremely shallow source in order to solve for the optimum deformation source parameters (Table 1). The FE model was built with a digital elevation model (DEM) of Stromboli with spatial resolution of 5 m, and a parametrized spherical cavity was created within the basaltic domain (Poisson coefficient 0.25 and density 2800 kg/m^3^). A tetrahedral mesh was used to discretize the domain, with a finer resolution (~ 2 m) around the cavity and the volcano summit. A constant pressure was applied to the internal cavity surface. The model was solved for stationary solution. The cavity parameters were obtained by a Monte Carlo algorithm with the objective to minimize the root mean square error between the measured tilt and the predicted one. Results were mostly in accordance with those obtained with half-space geometry. Monte Carlo results were also used to calculate associated uncertainties to model parameters.

## Supplementary Information

Supplementary Dataset File 1.

Supplementary Dataset File 2.

Supplementary Dataset File 3.

Supplementary Dataset File 4.

Supplementary Figure 1.

## Data Availability

All petrological data generated and analyzed during this study are included in this published article (and its supplementary files). The geophysical data that support the findings of this study are available from the INGV but restrictions apply to the availability of these data, which were used under license for the current study, and so are not publicly available. Data are however available from the authors upon reasonable request and with permission of the INGV.
