# Supplementary material for: Shallow conduit dynamics fuel the unexpected paroxysms of Stromboli volcano during the summer 2019
Source: Sci Rep. 2021 Jan 11;11:266. doi: 10.1038/s41598-020-79558-7 (PMC7801714; doi:10.1038/s41598-020-79558-7)

Shallow conduit dynamics fuel the unexpected paroxysms of Stromboli volcano during the summer 2019

Marco Viccaro[1,2*], Andrea Cannata[1,2], Flavio Cannavò[2], Rosanna De Rosa[3], Marisa Giuffrida[1], Eugenio Nicotra[3], Maurizio Petrelli[4], Gaia Sacco[1]

[1] *Università degli Studi di Catania, Dipartimento di Scienze Biologiche, Geologiche e Ambientali, Corso Italia 57, I-95129, Catania, Italy*

[2] *Istituto Nazionale di Geofisica e Vulcanologia – Sezione di Catania, Osservatorio Etneo, Piazza Roma 2, I-95125 Catania, Italy*

[3] *Università della Calabria, Dipartimento di Biologia, Ecologia e Scienze della Terra, Ponte Pietro Bucci, I-87036, Arcavacata di Rende, Italy*

[4] *Università degli Studi di Perugia, Dipartimento di Fisica e Geologia, Piazza dell'Università, Palazzo delle Scienze, I-06123, Perugia, Italy*

* Corresponding author

Supplementary Figure 1 - Seismograms of the vertical component of the signal recorded by STR1 during July 3, 2019 (a) and August 28, 2019 (b).

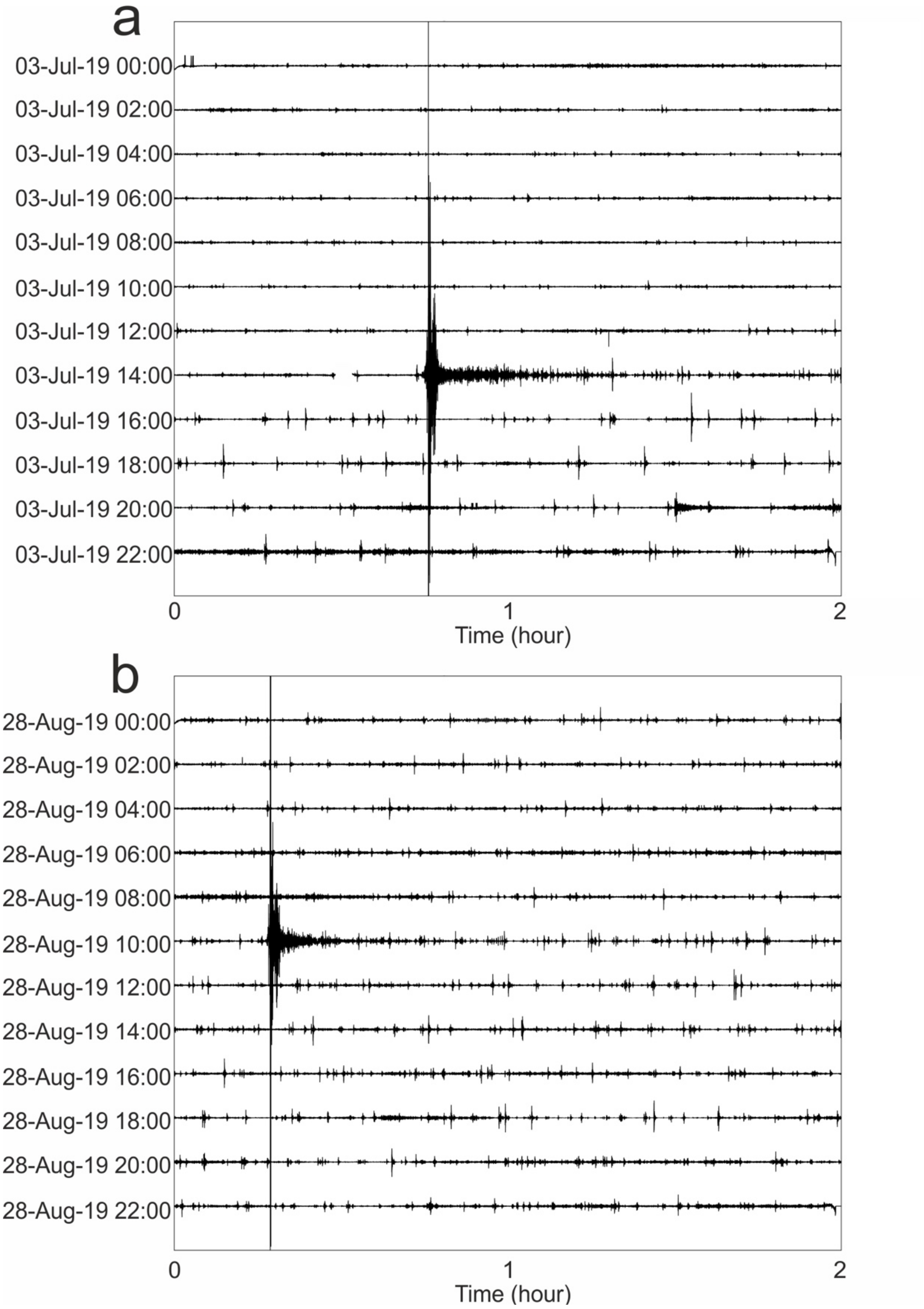

Supplement: Supplementary file 5 — Supplementary Figure 1. [file 41598_2020_79558_MOESM5_ESM.pdf]
